# Energy-Efficient Dynamic Enhanced Inter-Cell Interference Coordination Scheme Based on Deep Reinforcement Learning in H-CRAN

**DOI:** 10.3390/s24247980

**Published:** 2024-12-13

**Authors:** Hyungwoo Choi, Taehwa Kim, Seungjin Lee, Hoan-Suk Choi, Namhyun Yoo

**Affiliations:** 1 College of AI/SW Convergence, Kyungnam University, 7 Gyeongnamdaehak-ro, Masanhappo-gu, Changwon 51767, Republic of Korea; hwchoi@kyungnam.ac.kr (H.C.); sjinlee@kyungnam.ac.kr (S.L.); chs2024@kyungnam.ac.kr (H.-S.C.); hyun43@kyungnam.ac.kr (N.Y.); 2 School of Electrical Engineering, Korea Advanced Institute of Science and Technology (KAIST), 291 Daehak-ro, Yuseong-gu, Daejeon 34141, Republic of Korea

**Keywords:** 5G networks, Internet of Things, Cloud RAN, energy efficiency, interference coordination, reinforcement learning

## Abstract

The proliferation of 5G networks has revolutionized wireless communication by delivering enhanced speeds, ultra-low latency, and widespread connectivity. However, in heterogeneous cloud radio access networks (H-CRAN), efficiently managing inter-cell interference while ensuring energy conservation remains a critical challenge. This paper presents a novel energy-efficient, dynamic enhanced inter-cell interference coordination (eICIC) scheme based on deep reinforcement learning (DRL). Unlike conventional approaches that focus primarily on optimizing parameters such as almost blank subframe (ABS) ratios and bias offsets (BOs), our work introduces the transmission power during ABS subframes (TPA) and the channel quality indicator (CQI) threshold of victim user equipments (CTV) into the optimization process. Additionally, this approach uniquely integrates energy consumption into the scheme, addressing both performance and sustainability concerns. By modeling key factors such as signal-to-interference-plus-noise ratio (SINR) and service rates, we introduce the concept of energy-utility efficiency to balance energy savings with quality of service (QoS). Simulation results demonstrate that the proposed scheme achieves up to 70% energy savings while enhancing QoS satisfaction, showcasing its potential to significantly improve the efficiency and sustainability of future 5G H-CRAN deployments.

## 1. Introduction

The rapid evolution of mobile communication technologies has ushered in a new era of connectivity, with fifth-generation (5G) networks at the forefront of this transformation. Fifth-generation networks promise unprecedented speeds, ultra-low latency, and massive device connectivity, enabling a wide array of novel applications and services. At the same time, the industrial sector has witnessed a surge in the adoption of industrial internet of things (IIoT) technologies, revolutionizing manufacturing processes and operational efficiencies [1]. To address the unique demands of IIoT applications in industrial settings, many organizations are turning to private 5G networks. These dedicated networks offer enhanced security, reliability, and customization capabilities, making them ideal for supporting mission-critical IIoT deployments in smart factories and industrial campuses. Moreover, the advent of 5G is closely intertwined with several other emerging technological paradigms, including the internet of things (IoT), heterogeneous networks (HetNet), and cloud radio access networks (CRAN). These advanced networks enable real-time data collection, analysis, and control in industrial settings, facilitating smart manufacturing, predictive maintenance, and optimized supply chain management.

At the heart of this revolution lies CRAN, a key technology that underpins the capabilities of 5G networks. CRAN, or centralized-RAN, is a promising network architecture for future wireless systems, which separates the baseband unit (BBU) and remote radio head (RRH). A centralized BBU pool controls a number of distributed RRHs. CRAN architecture is energy efficient in that the BBU pool is managed in a centralized data center, and dense deployment of RRHs reduces the distance to user equipments (UEs) and transmission power. In addition, CRAN architecture is cost-efficient in that low-cost RRHs are deployed in the remote site, not the whole BS systems. Thus, CRAN architecture has many benefits in terms of CAPEX and OPEX.

The combination of CRAN and HetNets is a useful solution, as it allows for dense deployment of small base stations (SBSs) to increase network capacity in HetNets. This kind of system is named heterogeneous CRAN (H-CRAN) [2]. In H-CRAN, a macro basestation (MBS) controls signaling and takes charge of seamless coverage, and RRHs are in charge of high-speed data service in hotspot areas. The cost and energy-efficient advantages can be applied as it is by adopting the CRAN architecture in HetNets. Nevertheless, the load imbalance issues and inter-and intra-cell interference still need to be managed carefully.

The enhanced inter-cell interference coordination (eICIC) scheme is a prominent interference coordination technique in HetNets. The almost blank subframe (ABS) is a time-domain interference coordination method that mitigates MBS interference to SBSs by silencing or reducing MBS transmission power during designated subframes. Furthermore, cell range expansion (CRE) facilitates the offloading of MBS traffic by enabling more UEs connections to SBSs.

**Figure 1:**
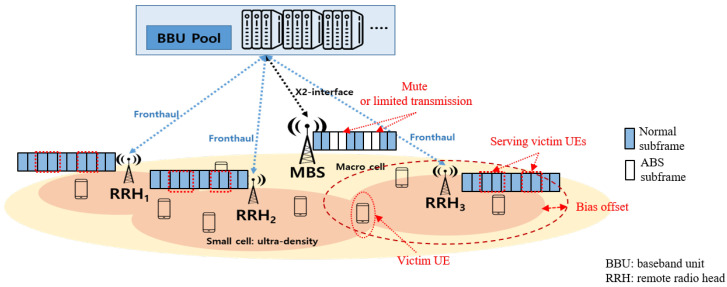
An example network layout of H-CRAN.

While previous studies on eICIC have focused on optimizing ABS and CRE parameters, such as ABS ratios and bias offsets (BOs) in CRE, there are additional parameters that significantly influence eICIC performance. These include the MBS transmission power during ABS subframes and the channel quality indicator (CQI) threshold of victim UEs. The MBS transmission power during ABS (TPA) subframes directly impacts the interference levels and the performance of UEs connected to the MBS. Similarly, the CQI threshold of victim UEs (CTV) determines how many victim UEs are served during ABS subframes, affecting the overall resource allocation. Thus, the joint optimization of the ABS ratio, BOs, TPA, and CTV is crucial for maximizing H-CRAN performance. Figure 1 depicts an example network layout of H-CRAN and the operation of eICIC parameters. To the best of our knowledge, this is the first attempt to consider TPA and CTV as optimizing parameters for interference coordination.

However, despite the energy efficiency gains of CRAN, the dense deployment of RRHs for small cells increases overall energy consumption. To address this, sleep mode control can be applied to deactivate underutilized RRHs when appropriate. Nonetheless, activating sleep modes changes network conditions, which forces UEs to reconnect to other active base stations, impacting interference patterns and UE quality of service (QoS). Therefore, a comprehensive approach that jointly optimizes eICIC, energy conservation, and UE QoS is essential for achieving optimal network performance.

In this paper, we propose an energy-efficient dynamic eICIC scheme based on deep reinforcement learning (DRL) in H-CRAN. The main contributions of this paper are summarized as follows:

- We propose a novel dynamic eICIC scheme tailored for H-CRAN. The scheme optimizes various interference coordination parameters and integrates energy efficiency, addressing the challenges specific to dense 5G environments.
- Unlike traditional eICIC studies that focus on parameters such as ABS ratios and BOs of CRE, this work extends the optimization scope by incorporating additional parameters, like TPA and CTV. These additional control dimensions enable more precise interference management and performance improvements.
- The proposed approach aims to balance energy efficiency and QoS. By introducing the concept of energy-utility efficiency (EU-efficiency), we jointly optimize the network’s service quality and energy consumption, a step forward in promoting sustainable 5G network operations.
- To enhance energy savings, our method integrates SBS sleep mode control into the optimization framework. By dynamically deactivating underutilized SBSs, the network achieves significant energy savings without sacrificing service quality.
- DRL is employed to optimize the multiparameter eICIC system, effectively tackling the curse of dimensionality that arises from simultaneously tuning multiple network parameters. The DRL agent navigates this complex high-dimensional decision space through continuous interaction with the network environment, addressing optimization challenges that would be intractable for conventional methods. This approach enables dynamic adaptation of eICIC parameters in response to varying network conditions, improving system robustness and performance.
- Through comprehensive simulations, the proposed scheme demonstrates substantial improvements in both energy savings (up to 70%) and QoS satisfaction. These results suggest that the approach is highly effective in real-world 5G deployments, particularly for dense urban scenarios and IoT use cases.

## 2. Related Works

HetNets are pivotal for addressing the growing complexities of 5G and beyond. They tackle key challenges such as interference management, resource allocation, and energy efficiency. By leveraging advanced methodologies, including machine learning, optimization techniques, and reinforcement learning, researchers have developed innovative solutions for the efficient and scalable operation of HetNets.

Interference management, load balancing, and resource allocation are critical for enhancing network performance, especially in dense deployments. In our previous work, we proposed a cooperative multiagent reinforcement learning-based scheme for BO control in CRE to optimize load balancing in dense HetNets [3]. Our method introduced a QoS satisfaction indicator, considering delay, data rate, and signal-to-interference-plus-noise ratio (SINR), and demonstrated significant improvements in throughput, delay satisfaction, and fairness under medium and high traffic loads. Ahmad et al. addressed co-channel interference by integrating eICIC and coordinated multipoint scheduling [4]. Their method demonstrated improved resource utilization for critical applications, such as public safety networks and UAV-based systems, by dynamically allocating radio resources and mitigating interference. Similarly, Sciancalepore et al. proposed a semi-distributed mechanism using ABS to optimize resource allocation in multitraffic scenarios [5]. Their framework balanced guaranteed traffic with best-effort throughput, improving overall network efficiency in dense urban environments. Xu et al. contributed to this area by employing a DRL-based framework for load balancing in ultra-dense networks [6]. Their method dynamically clustered small cells and distributed traffic adaptively, ensuring network stability under fluctuating traffic demands. Together, these studies highlight the importance of integrating interference coordination, load balancing, and resource allocation into cohesive, adaptable frameworks to address the dynamic challenges of modern networks.

Energy efficiency is increasingly vital for the sustainability of dense networks, with significant contributions focusing on reducing energy consumption while maintaining performance. Pan and Yang developed a DRL-based optimization framework for device-to-device communication, enabling dynamic adjustments in transmit power and spectrum allocation [7]. This approach achieved considerable energy savings while maintaining network stability, demonstrating the potential of real-time adaptive optimization techniques. Ayala-Romero et al. introduced an online learning algorithm that combined energy saving and interference coordination using a contextual bandit model [8]. Their framework dynamically activated small cells to reduce energy consumption without compromising QoS, especially in high-traffic environments. Wei et al. explored robust optimization frameworks for multicell interference exploitation under imperfect channel state information [9]. By maintaining user SINRs while minimizing transmission power, their method achieved both energy efficiency and interference mitigation. These studies underline the critical role of energy-efficient designs in sustaining scalable HetNet deployments.

Reinforcement learning has emerged as a powerful tool for addressing resource allocation, energy management, and information timeliness in HetNets. Zheng et al. introduced a distributed deep deterministic policy gradient (DDPG)-based algorithm to minimize the age of information in wireless-powered IoT systems [10]. Their hybrid framework combines discrete action selection from deep Q-networks (DQN) with continuous control capabilities of DDPG, optimized channel selection, transmission duration, and power allocation. This approach significantly reduced age of information and energy consumption, offering practical solutions for real-time communication scenarios requiring high timeliness. Tian et al. developed a multiagent DRL framework for vehicular networks, optimizing channel allocation and power control to meet the diverse requirements of delay-sensitive safety communications and high-bandwidth entertainment services [11]. Akyıldız et al. employed hierarchical reinforcement learning for resource allocation in radio access network slicing, ensuring efficient resource sharing between enhanced mobile boradband (eMBB) and ultra reliable and low latency communication (URLLC) slices while reducing computational overhead [12]. Huang et al. applied multiagent DRL to mobile edge computing, introducing a framework that optimized computation offloading and interference coordination [13]. Their approach minimized energy consumption and latency, demonstrating the synergy between RL and edge intelligence in improving network performance.

Automation and adaptability are increasingly integral to network optimization. Liu et al. used DRL to automate network configurations in the internet of vehicles, dynamically adapting to changing environments [14]. This approach improved QoS metrics such as latency and reliability, addressing the complexities of highly mobile scenarios. Jiang et al. introduced a Mixed-DDPG framework for multiplexing eMBB and URLLC in wireless-powered networks [15]. Their model optimized time, energy, and subcarrier allocation, achieving higher throughput and energy efficiency.

These contributions collectively emphasize the significance of unified frameworks that integrate interference management, resource allocation, energy efficiency, and reinforcement learning. By addressing the interconnected challenges of HetNets, these methodologies provide the foundation for intelligent, scalable, and adaptive wireless systems capable of meeting the stringent demands of next-generation networks.

## 3. System Description and Problem Formulation

In this section, we detail the control parameters of the eICIC mechanism and the energy consumption model for our proposed scheme. Additionally, we formulate the problem using the QSI presented in [3] and introduce an MDP model for the proposed scheme. We consider the downlink of two-tier HetNets, where a single macro cell is overlaid with small cells consisting of cloud BBUs and RRHs. Typically, a macro cell is equipped with a three-sector directional antenna. For convenience, we regard each 120-degree sector antenna as a separate MBS.

Let M={1,2,…,m,…,M} denote a set of BSs, where a MBS is overlaid with a set of SBSs S={1,2,…s,…S}, and let $U_{i}=\left\{1,2,\ldots ,u_{i},\ldots ,U_{i}\right\}$ denote a set of UEs served by BS *i*.

### 3.1. Enhanced Inter-Cell Interference Coordination Control Parameters

eICIC scheme consists of two representative techniques, which are ABS and CRE. We briefly review the techniques and describe the parameters for the techniques.

SBSs expand their coverage by adding a BO to the reference signal received power (RSRP) of SBSs using the CRE technique, allowing more UEs near the cell edge to connect. CRE offloads traffic from an MBS to SBSs. The UEs select the serving BS $b_{i}^{serve}$ based on the RSRP $p_{i}^{rsrp}$ and the BO $\delta_{i}$ of BS *i*. Let $U_{i}$ denote the set of UEs served by BS *i*, where i∈M∪S. The UEs select the serving BS based on the RSRP $p_{i}^{rsrp}$ and the BO $\delta_{i}$ of BS *i* as follows:

(1) $$b_{i}^{serve}=\underset{i\in M\cup S}{\mathrm{argmax}}\left(p_{i}^{rsrp}+\delta_{i}\right).$$

Thus, the UE association and the traffic load of each BS depend on the BO $\delta_{i}$, directly or indirectly.

However, the offloaded UEs suffer from cross-tier interference caused by an adjacent MBS. To mitigate this interference, ABS is configured in the MBS. The main idea behind the ABS technique is that the aggressive cell causing dominant inter-cell interference to victim cells avoids transmission in certain subframes, allowing the victim cells to transmit data to their UEs. ABS prevents an adjacent MBS from interfering with cell-edge UEs in SBSs by muting the MBS, a process referred to as zero-power ABS (ZP-ABS). This approach reduces interference from MBSs to SBSs but sacrifices the performance of UEs at the MBS. To address this trade-off, low-power ABS (LP-ABS) was proposed in 3GPP Release 11 as a compromise to ZP-ABS. In LP-ABS, the MBS transmits with limited power during ABS subframes, enabling UEs near the MBS to still have transmission opportunities in these subframes. However, it is crucial to carefully set the parameters of ABS, as they significantly affect network performance. The main parameters of ABS include the ratio of ABS subframes and the transmission power in ABS subframes.

We assume Rayleigh block fading for the subchannels and orthogonal frequency-division multiple access (OFDMA) systems. The channel fading coefficient follows a complex Gaussian distribution, CN(0,1). The additive white Gaussian noise (AWGN) samples are independent and identically distributed (i.i.d.) complex Gaussian random variables, also CN(0,1). The channel fading coefficient remains constant during the coherence time and changes to a new value at the start of the next coherence time.

In addition, we adopt the OFDMA system due to its significant advantages in addressing the challenges inherent in HetNets. HetNets consist of a variety of cell types working in harmony, making efficient resource allocation, interference management, and multiuser support crucial for optimal network performance. OFDMA plays a vital role in solving these issues by enabling efficient frequency resource allocation, dynamic resource scheduling, and enhanced spectrum efficiency. This makes it particularly suitable for the complex nature of HetNets, where diverse wireless environments must be managed. By using OFDMA, we ensure that the network resources are utilized to their fullest potential, thus enhancing both the overall performance of the network and the quality of user experience. The inclusion of OFDMA also allows for higher spectrum efficiency and better handling of interference, making it an essential technology for modern HetNet deployments.

The MBS configures ABS subframes with a ratio $\alpha_{m}$, which represents the proportion of ABS subframes. During these ABS subframes, the MBS can either mute (zero-power) or transmit with limited transmission power. The transmission power of the MBS is denoted as $p_{0}^{normal}$ for non-ABS subframes and $p_{0}^{abs}$ for ABS subframes. The SINR at UE *k*, served by MBS *m*, is given by:

(2) $$SINR_{mk}=\left\{\begin{array}{l} \frac{p_{m}^{normal}\cdot g_{mk}}{\underset{j\in S}{\Sigma }p_{j}\cdot g_{jk}+n_{0}}\;\mathrm{for}\;\mathrm{non}\text{-}\mathrm{ABS}\;\mathrm{subframe},\\ \frac{p_{m}^{abs}\cdot g_{mk}}{\underset{j\in S}{\Sigma }p_{j}\cdot g_{jk}+n_{0}}\;\mathrm{for}\;\mathrm{ABS}\;\mathrm{subframe}, \end{array}\right.$$

where $p_{j}$ is the transmission power of SBS *j*, $g_{jk}$ is the channel gain from SBS *j* to UE *k*, and $n_{0}$ is the noise power. If the UE *k* is served by SBS *s*, then the SINR is given by:

(3) $$SINR_{sk}=\left\{\begin{array}{ll} \frac{p_{s}\cdot g_{sk}}{\underset{j\in S,j\neq s}{\Sigma }p_{j}\cdot g_{jk}+p_{m}^{normal}\cdot g_{mk}+n_{0}} & \mathrm{for}\;\mathrm{non}\text{-}\mathrm{ABS}\;\mathrm{subframe},\\ \frac{p_{s}\cdot g_{sk}}{\underset{j\in S,j\neq s}{\Sigma }p_{j}\cdot g_{jk}+p_{m}^{abs}\cdot g_{mk}+n_{0}} & \mathrm{for}\;\mathrm{ABS}\;\mathrm{subframe}. \end{array}\right.$$

Without the eICIC scheme, generally, the instant service rate $R_{ik}$ of the UE *k*, which can be supported by the associated BS *i* in a frame, can be given as [16]:

(4) $$R_{ik}=\frac{WT_{f}}{MN}\overset{N}{\underset{n=1}{\Sigma }}s_{k,n}\log_{2}\left(1+SINR_{ik}\right),$$

where *W* is the spectral bandwidth, $T_{f}$ is the frame duration, *M* is the number of slots, *N* is the number of subchannels, and $s_{k,n}\in \left\{0,1\right\}$ is the service indicator that denotes whether UE *k* is scheduled in subchannel *n*. $s_{k,n}=1$ if UE *k* is served in subchannel *n*, and $s_{k,n}=0$ if not.

In this paper, we assume that UEs are scheduled in a round-robin fashion. Then, the instantaneous service rate $R_{ik}$ of UE *k* at BS *i*, as given by Equation (4), becomes:

(5) $$R_{ik}=\frac{WT_{f}}{|U_{i}|\cdot N}\overset{N}{\underset{n=1}{\Sigma }}s_{k,n}\log_{2}\left(1+SINR_{ik}\right)\;\left(\mathrm{bit}/\mathrm{frame}\right),$$

where $|U_{i}|$ is the cardinality of a set of serving UEs of BS *i*. In addition, the effective capacity (EC) of UE *k* served by BS *i* is given by [16]:

(6) $$C_{ik}^{E}\left(SINR_{ik},\theta_{k}\right)=-\frac{1}{\theta_{k}T_{f}}\log\left(E\left\{e^{-\theta_{k}R_{ik}}\right\}\right),$$

where $\theta_{k}$ is the QoS exponent of UE *k* and $\theta_{k}$ is expressed by a QoS triplet $\left(\lambda_{k},D_{k}^{max},\varepsilon_{k}\right)$, which represents the traffic attribute of UE with a traffic arrival rate $\lambda_{k}$, a maximum delay bound $D_{k}^{max}$, and a delay violation probability $\varepsilon_{k}$.

**Figure 2:**
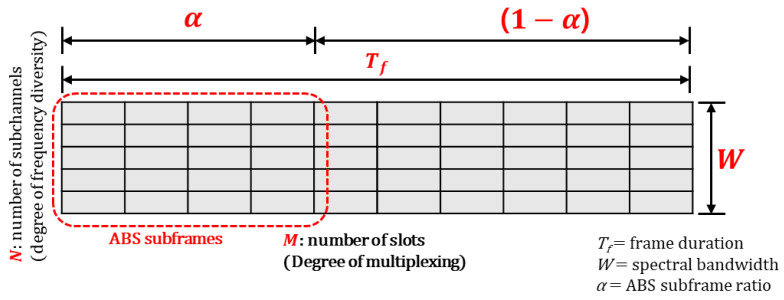
A conceptual representation of the ABS subframes in a frame structure of OFDMA.

With the eICIC scheme, the SINR of UEs is defined as Equations (2) and (3). Additionally, SBSs determine the victim UEs to be scheduled in ABS subframes by a victim threshold. SBSs select UEs whose CQI is below this victim threshold as victim UEs. Furthermore, as a time-domain ICIC scheme, ABS makes a MBS mute or transmit with a limited transmission power during α subframes and transmit with a normal transmission power during (1−α) subframes, as depicted in Figure 2. Then, from Equations (3) and (5) the instant service rate of the normal UE *k* at SBS *s* is given by:

(7) $$\begin{array}{cc} R_{sk}= & \frac{WT_{f}\cdot \left(1-\alpha \right)}{|U_{s}^{normal}|\cdot N}\overset{N}{\underset{n=1}{\Sigma }}s_{k,n}\log_{2}\left(1+SINR_{sk}\right),\\ = & \frac{WT_{f}\cdot \left(1-\alpha \right)}{|U_{s}^{normal}|\cdot N}\overset{N}{\underset{n=1}{\Sigma }}s_{k,n}\log_{2}\left(1+\frac{p_{s}\cdot g_{sk}}{\underset{j\in S,j\neq s}{\Sigma }p_{j}\cdot g_{jk}+p_{m}^{normal}\cdot g_{mk}+n_{0}}\right), \end{array}$$

where $|U_{s}^{normal}|$ is the number of normal UEs at BS *s* that satisfies $\left|U_{s}|=|U_{s}^{normal}|+|U_{s}^{victim}\right|$.

Also, from Equations (3) and (5) the instant service rate of the victim UE *k* at SBS *s* is given by:

(8) $$\begin{array}{cc} R_{sk}= & \frac{WT_{f}\cdot \alpha }{|U_{s}^{victim}|\cdot N}\overset{N}{\underset{n=1}{\Sigma }}s_{k,n}\log_{2}\left(1+SINR_{sk}\right),\\ = & \frac{WT_{f}\cdot \alpha }{|U_{s}^{victim}|\cdot N}\overset{N}{\underset{n=1}{\Sigma }}s_{k,n}\log_{2}\left(1+\frac{p_{s}\cdot g_{sk}}{\underset{j\in S,j\neq s}{\Sigma }p_{j}\cdot g_{jk}+p_{m}^{abs}\cdot g_{mk}+n_{0}}\right), \end{array}$$

where $|U_{i}^{victim}|$ is the number of victim UEs at BS *i* that satisfying $\left|U_{i}|=|U_{i}^{normal}|+|U_{i}^{victim}\right|$.

From Equations (2) and (5), the instant service rate of the UEs at the MBS is given by:

(9) $$\begin{array}{cc} R_{mk} & =\frac{WT_{f}}{|U_{m}|\cdot N}\overset{N}{\underset{n=1}{\Sigma }}s_{k,n}\log_{2}\left(1+SINR_{mk}\right),\\ & =\frac{WT_{f}\cdot \alpha }{|U_{m}|\cdot N}\overset{N}{\underset{n=1}{\Sigma }}s_{k,n}\log_{2}\left(1+\frac{p_{m}^{abs}\cdot g_{mk}}{\underset{j\in S}{\Sigma }p_{j}\cdot g_{jk}+n_{0}}\right)\\ & \;+\frac{WT_{f}\cdot \left(1-\alpha \right)}{|U_{m}|\cdot N}\overset{N}{\underset{n=1}{\Sigma }}s_{k,n}\log_{2}\left(1+\frac{p_{m}^{normal}\cdot g_{mk}}{\underset{j\in S}{\Sigma }p_{j}\cdot g_{jk}+n_{0}}\right), \end{array}$$

where $|U_{m}|$ is the number UEs at the MBS *m*.

The BO of SBSs affects $|U_{m}|$ and $|U_{s}|$, and the victim threshold affects $\left|U_{i}|=|U_{i}^{normal}|+|U_{i}^{victim}\right|$. The ABS subframe ratio changes Equations (7)–(9), and accordingly, the transmission power of MBSs during ABS subframes has an influence on Equations (7)–(9), not only on Equations (2) and (3). As a result, the instant service rate Equations (7)–(9) and the effective capacity Equation (6) of UEs in the network, depend on the eICIC parameters, such as ABS subframe ratio, MBS transmission power in ABS subframe, BO of SBSs for CRE, and victim threshold at SBSs.

### 3.2. Base Station Power Consumption Model

In this subsection, we present the power consumption model of BSs based on 3GPP guidelines [17]. The basic assumption is that the power consumption of BSs is linear to the RF output power, and depends on the load of BSs. The power consumption model of MBS defined as follows:

(10) $$\begin{array}{c} e_{m}=\left\{\begin{array}{cc} N_{TRX}\cdot (P_{m}^{0}+\Delta p_{m}\cdot p_{m}^{normal}),\;0<p_{m}^{normal}<p_{m}^{max}\; & \mathrm{for}\;\mathrm{non}\text{-}\mathrm{ABS}\;\mathrm{subframe},\\ N_{TRX}\cdot \left(P_{m}^{0}+\Delta p_{m}\cdot p_{m}^{abs}\right),0<p_{m}^{abs}<p_{m}^{max}\; & \mathrm{for}\;\mathrm{ABS}\;\mathrm{subframe}, \end{array}\right. \end{array}$$

where $N_{TRX}$ is the number of transceivers (TRXs) of MBS, $P_{m}^{0}$ is the basic power consumption of the TRX when the MBS is not transmitting, $\Delta p_{m}$ is the load factor of MBS, $p_{m}^{normal}$ is the transmission power of MBS at non-ABS subframes, and $p_{m}^{abs}$ is the transmission power of MBS at ABS subframes. According to the ABS ratio of MBS, Equation (10) can be rewritten by:

(11) $$e_{m}=\alpha_{m}\cdot N_{TRX}\cdot \left(P_{m}^{0}+\Delta p_{m}\cdot p_{m}^{abs}\right)+\left(1-\alpha_{m}\right)\cdot N_{TRX}\cdot \left(P_{m}^{0}+\Delta p_{m}\cdot p_{m}^{normal}\right).$$

The power consumption model of SBS is defined as follows:

(12) $$\begin{array}{c} e_{s}=\left\{\begin{array}{cc} N_{TRX}\cdot \left(P_{s}^{0}+\Delta p_{s}\cdot p_{s}\right),0<p_{s}^{normal}<p_{s}^{max}\;\; & \;\;\mathrm{for}\;\mathrm{nonsleep}\;\mathrm{state},\\ N_{TRX}\cdot P_{s}^{sleep}\;\;\;\;\;\;\; & \;\;\mathrm{for}\;\mathrm{sleep}\;\mathrm{state}, \end{array}\right. \end{array}$$

where $N_{TRX}$ is the number of TRXs of SBS, $P_{s}^{0}$ is the basic power consumption of the TRX when the SBS is not transmitting, $\Delta p_{s}$ is the load factor of SBS, $p_{s}$ is the transmission power of SBS, and $P_{s}^{sleep}$ is the power consumption of TRXs when the SBS is in sleep mode. We assume that SBS can switch between active mode and sleep mode, and Equation (12) can be rewritten by:

(13) $$e_{s}=\epsilon_{s}\cdot N_{TRX}\cdot \left(P_{s}^{0}+\Delta p_{s}\cdot p_{s}\right)+\left(1-\epsilon_{s}\right)\cdot N_{TRX}\cdot P_{s}^{sleep},$$

where $\epsilon_{s}=2$ if the SBS *s* is in active mode and $\epsilon_{s}=0$ if the SBS *s* is in sleep mode. Table 1 shows the power model parameters that are presented in the recommendation of 3GPP [17]. In this paper, we assume that $N_{TRX}$ is 6 for MBS and 2 for SBS as the recommendation of 3GPP [17].

### 3.3. Energy-Utility Efficiency

In our previous work [3], we have proposed the QSI utility function for BS, which measures both qualitative and quantitative statistics UEs’ QoS satisfaction. The QSI utility function of BS *i* where i∈M∪S is defined as follows:

(14) $$\psi_{i}\left(s^{t},a_{i}^{t}\right)=\mu_{i}\left(s^{t},a_{i}^{t}\right)-\frac{\rho }{2}\sigma_{i}^{2}\left(s^{t},a_{i}^{t}\right),$$

where $\mu_{i}\left(\cdot \right)$ is the mean of QSI value, $\sigma_{i}\left(\cdot \right)$ is the variance of the QSI value of BS *i*, and ρ>0 is the risk aversion parameter, which is is a foundational principle of risk-aware rational decision making [18]. We assume that the eICIC parameters are remain constant during the time slot *t*. The QSI utility depends on the states and the eICIC parameters of BSs at time slot *t*. Detailed definitions of $s^{t},a_{i}^{t}$ are presented in the next subsection.

The QSI values means the UE’s QoS satisfaction, which is calculated by the ratio of the actual measured value to the effective capacity as follows:

(15) $$f_{ik}=\frac{1}{h}\overset{h}{\underset{j=1}{\Sigma }}\frac{C_{ik,j}^{M}}{C_{ik,j}^{E}\left(SINR_{ik,j},\theta_{ik}\right)},$$

where $C_{ij,j}^{M}$ is the measured capacity, $C_{ik,j}^{E}\left(\cdot \right)$ is the effective capacity of UE *k* at *j*-th frame during *h* frames, $SINR_{ik,j}$ is the SINR of UE *k* at *j*-th frame of BS *i*, and $\theta_{ik}$ is the QoS exponent of UE *k* at BS *i*. For more detailed information, please refer to the paper [3].

In this paper, we also use the QSI utility function Equation (14) to represent the utility in terms of the QoS satisfaction ratio of BSs. In this section, we define EU-efficiency to consider both energy efficiency and QSI utility together. The EU-efficiency is defined as:

(16) $$\eta \left(s^{t},a_{m}^{t},a_{s}^{t}\right)=\frac{{\left\{\underset{m\in M}{\Sigma }\psi_{m}\left(s^{t},a_{m}^{t}\right)+\underset{s\in S}{\Sigma }\psi_{s}\left(s^{t},a_{s}^{t}\right)\right\}}^{\left(1-\xi \right)}}{{\left\{\underset{m\in M}{\Sigma }e_{m}\left(s^{t},a_{m}^{t}\right)+\underset{s\in S}{\Sigma }e_{s}\left(s^{t},a_{s}^{t}\right)\right\}}^{\xi }},$$

where $\psi_{m}\left(\cdot \right)$ and $\psi_{s}\left(\cdot \right)$ are the utility from Equation (14) and $e_{m}\left(\cdot \right)$ and $e_{s}\left(\cdot \right)$ are the energy consumption of MBS and SBS, respectively, which are calculated by Equations (11) and (13). The parameter ξ is an energy sensitivity parameter, where 0≤ξ≤1. When ξ approaches 1, more emphasis is placed on energy consumption in η(·), and when ξ approaches 0, more emphasis is placed on utility in η(·). In our system, the action $a_{i}^{t}$ differs for MBS and SBS, which are represented by $a_{m}^{t}$ and $a_{s}^{t}$, respectively. In fact, $a_{m}^{t}=\left(\alpha^{t},\beta^{t}\right)$, the vector of eICIC parameters for MBSs, includes the ABS ratio ($\alpha^{t}=\left[\alpha_{1}^{t},\ldots ,\alpha_{m}^{t}\right]$) and the MBS transmission power during the ABS subframe ($\beta^{t}=\left[\beta_{1}^{t},\ldots ,\beta_{m}^{t}\right]$). Additionally, $a_{s}^{t}=\left(\gamma^{t},\delta^{t},\epsilon^{t}\right)$, the vector of eICIC parameters and sleep modes for SBSs, includes the victim threshold of SBSs $\gamma^{t}=\left[\gamma_{1}^{t},\ldots ,\gamma_{s}^{t}\right]$, the BOs of SBSs $\delta^{t}=\left[\delta_{1}^{t},\ldots ,\delta_{s}^{t}\right]$, and the sleep mode of SBSs $\epsilon^{t}=\left[\epsilon_{1}^{t},\ldots ,\epsilon_{s}^{t}\right]$.

Our objective is the maximization of Equation (16), and we need to find the optimal eICIC parameters for MBSs and SBSs as:

(17) $$\alpha^{t},\beta^{t},\gamma^{t},\delta^{t},\epsilon^{t}=\underset{\alpha^{t},\beta^{t},\gamma^{t},\delta^{t},\epsilon^{t}}{\mathrm{argmax}}\eta \left(\cdot \right).$$

### 3.4. Markov Decision Model for Deep Q-Networks

We model the RL problem in the form of an MDP, a mathematical framework for modeling decision making. It consists of 4-tuple (S,A,P,R), where *S* is a finite set of states, *A* is a finite set of actions, *P* is a state transition probability, and *R* is a reward function.

#### 3.4.1. State

We define the state of BS *i* is a joint vector of $I_{\mu }$ and $I_{\sigma }$:

(18) $$s_{i}=\left(I_{\mu_{i}},I_{\sigma_{i}^{2}}\right),$$

where

$$\begin{array}{cc} \; & I_{\mu_{i}}=\left\{\begin{array}{cc} 0, & \;\mathrm{if}\;\mu_{i}<1-\omega_{\mu },\\ 1, & \;\mathrm{if}\;1-\omega_{\mu }\leq \mu_{i}\leq 1+\omega_{\mu },\\ 2, & \;\mathrm{if}\;\mu_{i}>1+\omega_{\mu }, \end{array}\right.\\ \; & I_{\sigma_{i}^{2}}=\left\{\begin{array}{cc} 0, & \;\mathrm{if}\;\sigma_{i}^{2}\leq \omega_{\sigma^{2}},\\ 1, & \;\mathrm{if}\;\sigma_{i}^{2}>\omega_{\sigma^{2}}, \end{array}\right. \end{array}$$

and $\omega_{\mu }$ and $\omega_{\sigma^{2}}$ are the tuning parameters for determining the optimality of BSs. The value $I_{\mu_{i}}$ indicates the average delay satisfaction level of UEs at BS *i*. An $I_{\mu_{i}}$ of 0 means that UEs at BS *i* receive poor service on average, while an $I_{\mu_{i}}$ of 2 indicates that UEs receive excellent service. An $I_{\mu_{i}}$ of 1 reflects adequate service. Factors influencing $I_{\mu_{i}}$ may include the SINR of UEs or the density of UEs at BS *i*, which can lead to resource competition. The index $I_{\sigma_{i}^{2}}$ represents the load balance, indicating whether the load is balanced in terms of the variance of QSI. The variance of QSI reflects fairness among UEs in the cell. When all UEs in the cell meet their QoS requirements at similar levels, the QSI variance approaches zero, indicating a more balanced state ($I_{\sigma_{i}^{2}}=0$). Therefore, the combination of $I_{\mu_{i}}$ and $I_{\sigma_{i}^{2}}$ can represent a total of six possible states for BS *i*, with the state (1,0) being the most well-balanced. The overall state of the network at time slot *t* is then represented as:

(19) $$s^{t}=\left[s_{1}^{t},\ldots ,s_{i}^{t}\right],\forall i\in M\cup S.$$

#### 3.4.2. Action

- *ABS ratio:* We denote the ABS ratio of MBS *m* as $\alpha_{m}$, which can take values from the set of possible ABS ratios $\alpha_{m}^{1},\alpha_{m}^{2},\ldots ,\alpha_{m}^{k},m\in M$. For example, in time division duplex (TDD) mode, the ABS patterns with muting ratios could be 1/10, 2/10, and so on, since there are 10 subframes, each with a duration of 1 ms, within a 10 ms frame. Therefore, the action space for the ABS ratio of an MBS can be 1/10,2/10,…,9/10.
- *Transmission power during ABS subframes:* We denote the transmission power of MBS *m* during ABS subframes as $\beta_{m}$. The possible transmission power levels during ABS subframes for MBS *m* are defined as $\beta_{m}^{1},\beta_{m}^{2},\ldots ,\beta_{m}^{l},m\in M$. For example, the transmission power during ABS subframes can be expressed as a ratio of the normal transmission power. Thus, the action space for the transmission power during ABS subframes of an MBS can be 0,0.05,0.1,…,0.5.
- *Victim UE CQI threshold:* We denote the CQI threshold for victim UEs at SBS *s* as $\gamma_{s}$. The possible thresholds at SBS *s* are defined as $\gamma_{s}^{1},\gamma_{s}^{2},\ldots ,\gamma_{s}^{q},s\in S$. In general, the CQI provides information about the highest modulation and coding scheme (MCS) suitable for downlink transmission to achieve the required block error rate (BLER) under given channel conditions. The CQI index ranges from 1 to 15. Therefore, the action space for the CQI threshold of victim UEs can be 1,2,…,15.
- *Bias offset for CRE:* We denote the BO of SBS *s* as $\delta_{s}$, with the possible BO values defined as $\delta_{s}^{1},\delta_{s}^{2},\ldots ,\delta_{s}^{r},s\in S$. For example, if the BO of SBS *s* varies from 0 dB to 16 dB, then the action space for the BO can be 0,1,…,16.
- *Sleep mode switching:* We denote the sleep mode of SBS *s* as $\epsilon_{s}$, with its possible states being ‘on’ and ‘off’, represented as $\epsilon_{s}^{0},\epsilon_{s}^{1}$. If SBS *s* is in sleep mode, then $\epsilon_{s}^{0}=0$, and if it is in operating mode, then $\epsilon_{s}^{1}=1$. Therefore, the action space for sleep mode switching of an SBS is 0,1.

With the actions described above, the MBS *m* choose the action $a_{m}^{t}=\left[\alpha_{m}^{t},\beta_{m}^{t}\right]$ and the SBS *s* choose the action $a_{s}^{t}=\left[\epsilon_{s}^{t},\gamma_{s}^{t},\delta_{s}^{t}\right]$. The action of all MBSs is represented as $a_{m}^{t}=\left[a_{1},\ldots ,a_{m}\right],m\in M$. The action of all SBSs is represented as $a_{s}^{t}=\left[a_{1},\ldots ,a_{s}\right],s\in S$.

#### 3.4.3. State Transition Probability

State transition probabilities are challenging to model because they depend on various factors in real-world dynamics, such as UE mobility, channel conditions, and data rates. However, RL can solve the MDP without explicitly specifying the state transition probabilities, using a trial-and-error approach. Therefore, we adopt an RL-based algorithm to solve this MDP, which we will explain in detail in the next section.

#### 3.4.4. Reward

We define energy-utility efficiency Equation (16) as the reward for the network. The energy-utility efficiency consists of the sum of the utility and energy consumption for each MBS and SBS. Each BS applies its actions individually, and the utility and energy consumption data are gathered at the central BBU to determine the reward for the actions. Thus, the reward is given as:

(20) $$\begin{array}{cc} R(s^{t},a_{m}^{t},a_{s}^{t}) & =\eta (s^{t},a_{m}^{t},a_{s}^{t}),\\ & =\frac{{\left\{\underset{m\in M}{\Sigma }\psi_{m}\left(s^{t},a_{m}^{t}\right)+\underset{s\in S}{\Sigma }\psi_{s}\left(s^{t},a_{s}^{t}\right)\right\}}^{\left(1-\xi \right)}}{{\left\{\underset{m\in M}{\Sigma }e_{m}\left(s^{t},a_{m}^{t}\right)+\underset{s\in S}{\Sigma }e_{s}\left(s^{t},a_{s}^{t}\right)\right\}}^{\xi }}. \end{array}$$

## 4. Deep Q-Networks for Energy-Utility Efficient Dynamic Enhanced Inter-Cell Interference Coordination

Applying conventional reinforcement learning algorithms, such as Q-learning, to the parameter setting of eICIC in H-CRAN is challenging due to the high dimensionality of the action spaces. Therefore, we utilize DQN to determine the optimal eICIC parameters. Additionally, we assume that the DQN agents are deployed at the central BBU.

**Figure 3:**
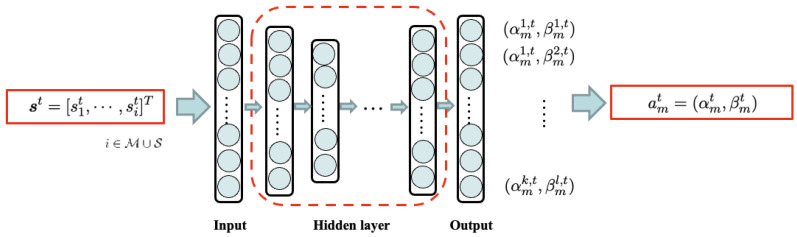
The structure of neural networks for MBS.

**Figure 4:**
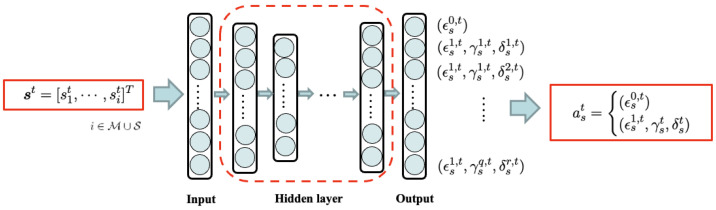
The structure of neural networks for SBS.

There are multiple BSs in the network, and each BS is assigned its own dedicated DQN agent to learn and execute its optimal actions. Although all DQN agents share the same state vector, which represents the overall state of the entire network, each DQN agent operates within a unique action space tailored to its specific BS type (MBS or SBS). The energy-utility efficiency of each BS contributes to the common network goal, as defined in Equation (20). To ensure that all agents select actions that collectively maximize the network’s energy-utility efficiency, we deploy a separate DQN agent for each BS. Each DQN agent learns to select the optimal action that maximizes the reward function in Equation (20). Figure 3 and Figure 4 illustrate the neural network structures for MBSs and SBSs, respectively.

**Figure 5:**
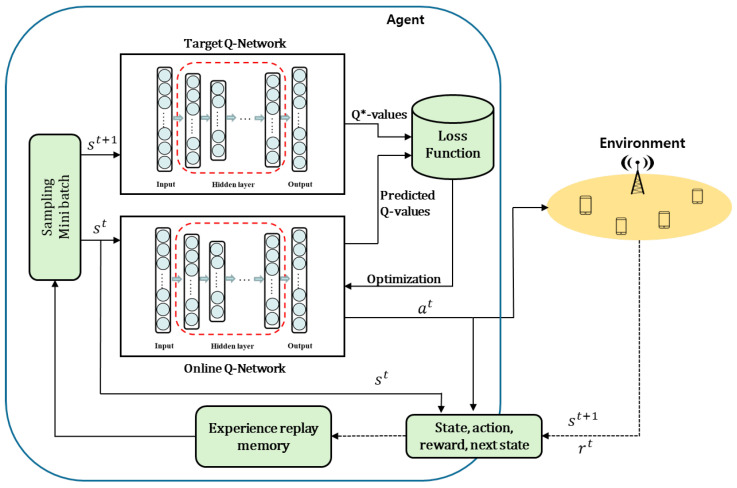
Double deep Q network.

We utilize double DQN (DDQN) [19] for our scheme because DQN [20] tends to overestimate action values, and DDQN is commonly used in most studies to address this issue. In general, the target value of DDQN can be expressed as:

(21) $$y^{t}=R^{t+1}+\rho Q\left(s^{t+1},\underset{a^{t}}{argmax}Q\left(s^{t+1},a^{t};\pi_{online}^{t}\right);\pi_{target}^{t}\right),$$

where ρ is the discount factor that ρ∈[0,1] and $\pi_{online}^{t}$ and $\pi_{target}^{t}$ are the vector of the weights of the neural networks for the online Q-network and the target Q-network, respectively. The DDQN is trained to minimize the loss function, which is given as:

(22) $$L\left(\pi_{online}^{t}\right)=E\left[{\left(y^{t}-Q\left(s^{t},a^{t};\pi_{online}\right)\right)}^{2}\right],$$

where $y^{t}$ is the target value from the target Q-network, and the target Q-network is updated per every some steps. The DDQN for BS *i* is depicted in Figure 5.

In this study, we selected the DQN approach due to its suitability for addressing problems with discrete state and action spaces, which aligns with the specific requirements of our system. While more advanced DRL algorithms, such as DDPG, proximal policy optimization (PPO), and twin delayed deep deterministic policy gradient (TD3), might offer improved performance in certain scenarios, the focus of this work is not on the specific algorithm itself. Instead, the core contribution lies in the formulation of the problem and the application of DRL to handle the complex input and output dynamics of the system. For the purposes of basic validation and demonstration of our approach, we employed the DDQN algorithm. However, any DRL algorithm designed for discrete state and action spaces could be utilized in this framework, making the approach flexible and adaptable.

**Figure 6:**
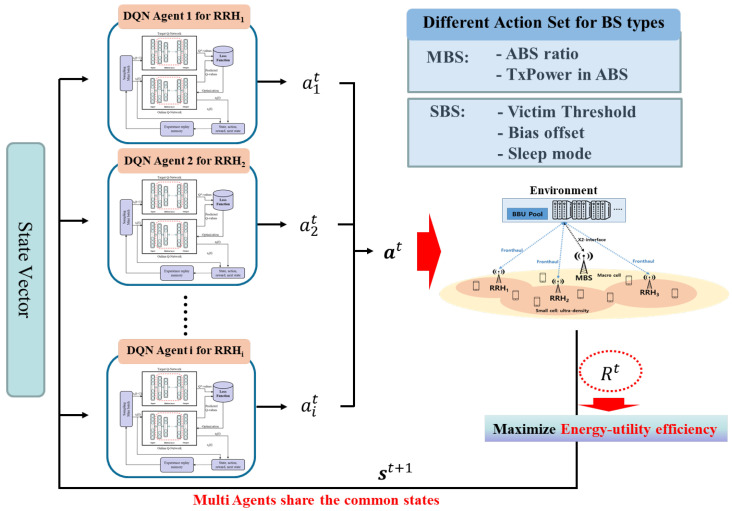
The abstract diagram of the overall system.

In a multiagent system where all agents share a common environment, we use the state vector of all BSs as a common input, and the reward Equation (20) is defined as the sum of the energy-utility efficiency of all BSs. This approach allows each BS to access global network information and learn the optimal actions for each BS. The centralized control feature of H-CRAN, which facilitates easy handover management without signaling overhead, makes this setting feasible. Figure 6 presents an abstract diagram of the overall system, and the detailed DDQN-based algorithm for our scheme is outlined in Algorithm 1.

**Algorithm 1** DDQN-based eICIC parameter and sleep mode learning

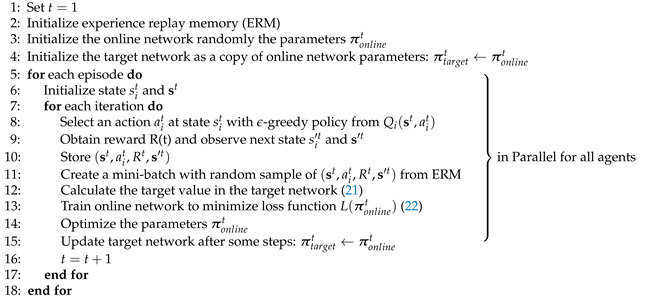



## 5. Performance Evaluation

**Figure 7:**
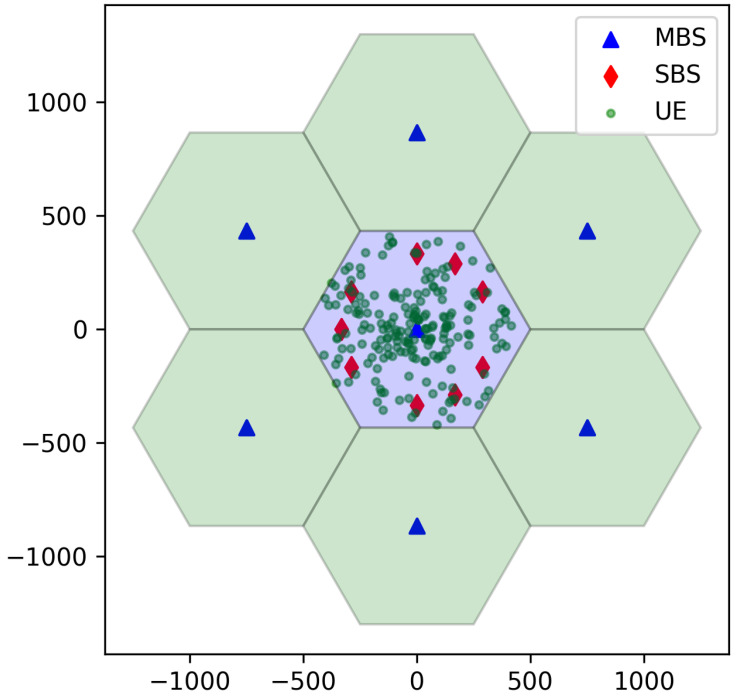
An example network layout for the simulations.

The performance evaluation was conducted using simulations developed based on the 3GPP guidelines for evaluating LTE networks [21]. The simulator was implemented in Python 3.7, utilizing the PyTorch 2.0 library for the DDQN algorithm. The network layout consists of 7 sectorized MBSs, each covering 120 degrees. SBSs are overlaid only in the central macro cell, while the other MBSs function solely as interferers. An example of the network layout is depicted in Figure 7.

UEs are randomly deployed in the central macro cell. We assume the QoS requirements for each UE as follows: an arrival rate of 300 kbps, a maximum delay bound of 150 ms, and a delay violation probability of 0.01. The sets of available parameters for eICIC are $\alpha_{m}=1/10,2/10,\ldots ,9/10$, $\beta_{m}=0,0.05,0.1,\ldots ,0.5$, $\gamma_{s}=1,2,\ldots ,15$, $\delta_{s}=0,1,\ldots ,16$, and $\epsilon_{s}=0,1$. The detailed simulation parameters are presented in Table 2. We evaluated the performance based on the number of UEs and the number of SBSs. The performance of our algorithm is compared across four different scenarios, each designed to evaluate specific aspects of network efficiency and produce reasonable results:

1. ***Default:*** In this baseline scenario, there is no eICIC operation. All SBSs are fully active and never switch to sleep mode, regardless of traffic conditions. This scenario helps evaluate the impact of not using any interference coordination or energy-saving strategies.
2. ***Fixed ABS-CRE***: In this scenario, MBSs operate with a fixed ABS subframe ratio, while SBSs use fixed CRE offsets. All SBSs remain active and never switch to sleep mode. This scenario serves as a control to understand the effects of static eICIC configurations on network performance.
3. ***DQN for ABS-CRE***: In this dynamic scenario, DQN agents adjust the ABS ratio for MBSs and the CRE BOs for SBSs based on real-time network conditions. However, all SBSs remain active and do not switch to sleep mode. This scenario evaluates the effectiveness of using machine learning to optimize only the traditional eICIC parameters (ABS and CRE) without considering energy-saving mechanisms.
4. ***DQN for ALL***: This is the proposed scenario, where DQN agents optimize not only the ABS subframe ratio and CRE BOs but also additional parameters such as TPA, CTV, and the sleep mode of SBSs. This scenario demonstrates the full potential of DQN in optimizing both network performance and energy efficiency, making it the most comprehensive and advanced of the four scenarios.

**Figure 8:**
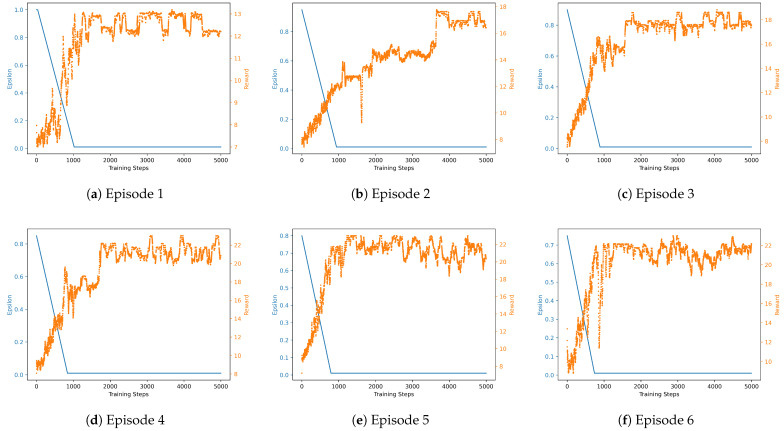
An example of the reward per episodes in the learning process of DDQN. The orange line represents the learning curve, and the blue line represents the exploration rate.

The convergence of the DDQN is illustrated in Figure 8. As the number of episodes increases, the DDQN progressively converges, ultimately achieving higher rewards. This demonstrates the model’s ability to learn and optimize its performance over time, leading to improved decision making and better outcomes.

**Figure 9:**
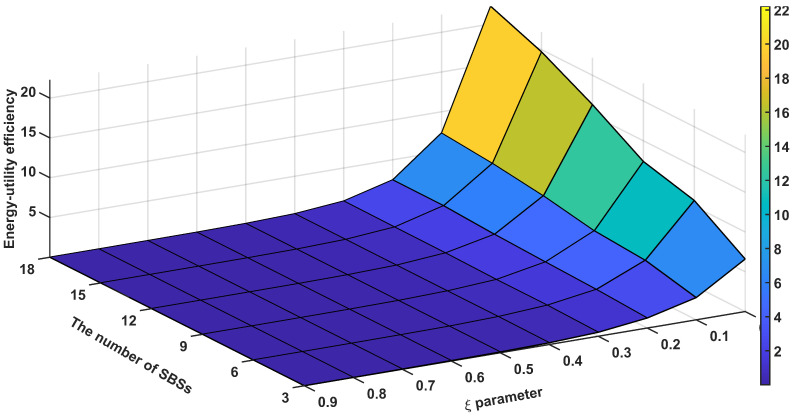
Average energy-utility efficiency performance according to the energy sensitivity parameter (ξ) and the number of SBSs.

Figure 9 presents the energy-utility efficiency, as defined in Equation (16), as a function of the weight ξ for different numbers of SBSs with 200 UEs deployed in the network. A larger value of ξ places greater emphasis on energy consumption in the energy-utility efficiency metric. Consequently, as ξ increases, the energy-utility efficiency decreases, and vice versa. When ξ=0, the energy-utility efficiency increases with the number of SBSs. Since ξ=0 indicates that the metric only considers QSI utility, the algorithm disregards energy consumption. As a result, no SBSs enter sleep mode, and the QSI utility improves as the number of SBSs increases.

**Figure 10:**
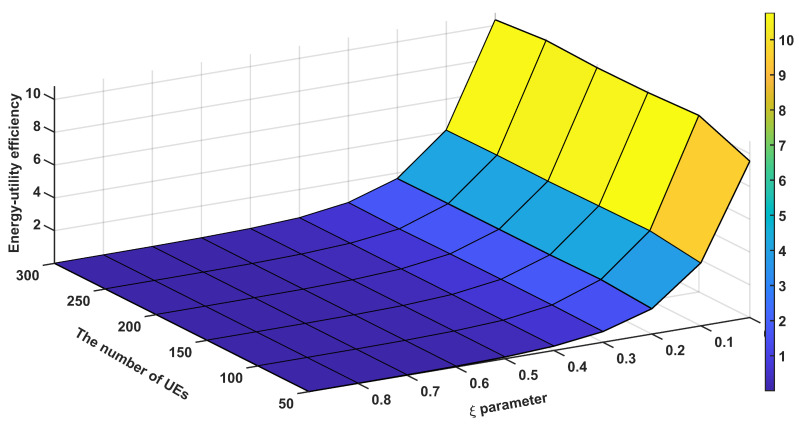
Average energy-utility efficiency performance according to energy sensitivity parameter (ξ) and the number of UEs.

Figure 10 presents the energy-utility efficiency as a function of the weight ξ for different numbers of UEs with 6 SBSs deployed in the network. Similar to Figure 9, the energy-utility efficiency increases as ξ decreases. The energy-utility efficiency remains relatively consistent regardless of the number of UEs. When ξ=0, the metric considers only QSI utility. In the case of 50 UEs, one might expect higher QSI utility due to the relatively abundant network resources. However, the graph shows a slightly lower value compared to other cases. This discrepancy can be attributed to the random deployment of UEs; with fewer UEs, they are more likely to be spread out, leading to a higher probability of some UEs being located in areas with poor SINR. These UEs in poor SINR regions are less effectively covered by eICIC, which in turn has a more significant impact on degrading QSI utility.

**Figure 11:**
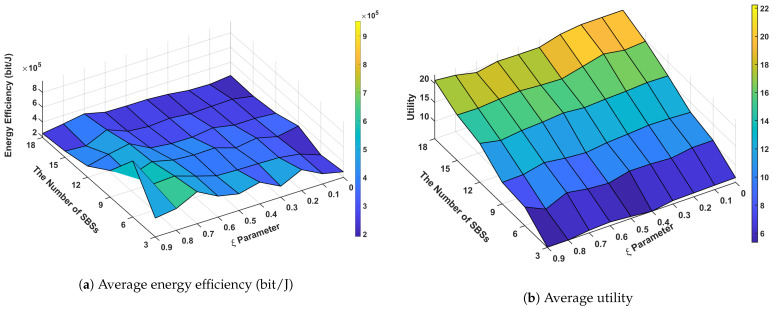
Performance according to energy sensitivity parameter (ξ) and the number of SBSs.

Figure 11 demonstrates the relationship between the ξ parameter, the number of SBSs, system utility, and energy efficiency in our proposed network architecture with 200 UEs deployed in the network. Energy efficiency is calculated by dividing the sum of all UE throughputs by the total network energy consumption in our simulations. The analysis reveals several significant findings regarding system performance optimization. The system utility shows a strong positive correlation with lower ξ values (0.1–0.3), reaching peak performance of 20–22 units with higher SBS densities (15–18 SBSs). This indicates that network utility is optimized when the system prioritizes resource allocation with smaller ξ parameters. In contrast, energy efficiency exhibits optimal performance (5–6 × 10^5^ bit/J) at higher ξ values (0.7–0.9), particularly evident in scenarios with moderate SBS deployment (9–12 SBSs). The correlation analysis between the ξ parameter and performance metrics reveals an inherent trade-off. As ξ increases from 0.1 to 0.9, system utility demonstrates a negative correlation, decreasing from peak values (20–22) to lower ranges (6–8). Conversely, energy efficiency shows a positive correlation with increasing ξ values, improving from 2 to 3 × 10^5^ to 5–6 × 10^5^ bit/J. This inverse relationship suggests that the ξ parameter effectively controls the balance between network performance and energy efficiency. A notable observation is the impact of SBS density on system performance. Higher numbers of SBSs (15–18) consistently yield better utility scores across all ξ values, demonstrating the system’s scalability. However, energy efficiency shows optimal performance in moderate SBS deployment scenarios, suggesting that excessive network densification may not necessarily lead to improved energy efficiency. The results validate our approach’s effectiveness in managing the performance-efficiency trade-off through ξ parameter optimization.

**Figure 12:**
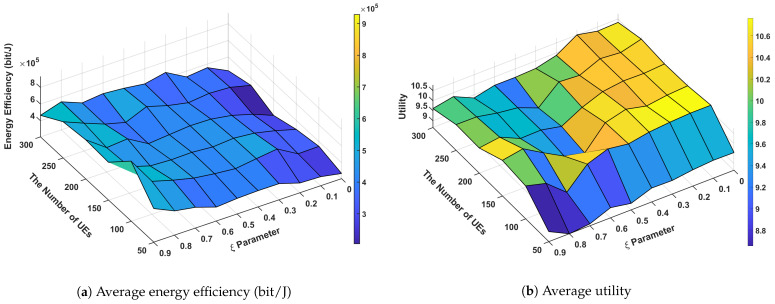
Performance according to energy sensitivity parameter (ξ) and the number of UEs.

Figure 12 demonstrates the performance characteristics of our proposed system across varying ξ parameters and UE densities with six SBSs deployed in the network. The analysis reveals significant findings in terms of system utility and energy efficiency metrics. Our system achieves peak utility scores of 10.4–10.6 in the lower ξ parameter range (0.1–0.3), particularly evident with higher UE densities (250–300). Conversely, energy efficiency reaches its optimal performance (7–9 × 10^5^ bit/J) at higher ξ values (0.7–0.9), highlighting a clear performance trade-off in the system design. The correlation analysis between ξ parameter and performance metrics reveals distinct patterns. As ξ increases from 0.1 to 0.9, utility shows a negative correlation, decreasing from peak values (10.4–10.6) to lower ranges (9.0–9.4). In contrast, energy efficiency demonstrates a positive correlation with increasing ξ values, showing marked improvement from 3 to 4 × 10^5^ to 7–9 × 10^5^ bit/J. This inverse relationship between utility and energy efficiency suggests that the ξ parameter effectively controls the system’s performance-efficiency balance. The utility metric shows positive correlation with UE density, indicating robust scalability of our approach. However, energy efficiency remains relatively consistent across different UE counts, suggesting that the system maintains stable power consumption characteristics despite varying network loads. A notable observation is the existence of a balanced operating point at moderate ξ values (0.4–0.6), where the system maintains acceptable utility (approximately 9.8–10.0) while preserving reasonable energy efficiency. This finding has practical implications for real-world deployments where both performance metrics are crucial. These results validate our approach’s effectiveness in managing the inherent trade-off between system utility and energy efficiency. The observed performance characteristics provide valuable insights for parameter optimization in practical network deployments, particularly in scenarios requiring dynamic adaptation to varying network conditions.

**Figure 13:**
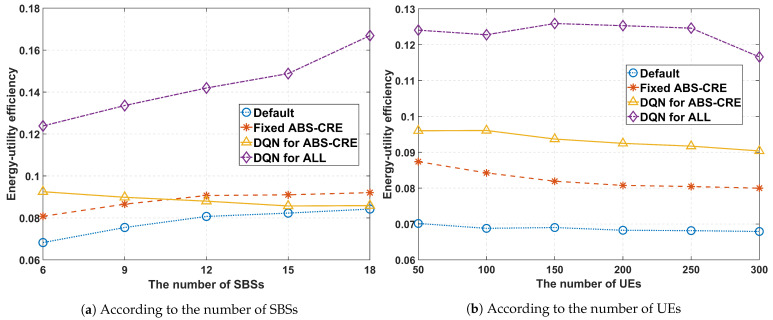
Average energy-utility efficiency performance.

Figure 13a compares the energy-utility efficiency of the proposed scheme with other schemes as the number of SBSs increases. The experiment was conducted with 200 UEs and ξ=0.5. For the proposed method, energy-utility efficiency increases as the number of SBSs grows, primarily because only the proposed scheme incorporates SBS sleep mode. This leads to a different trend in energy-utility efficiency compared to other schemes. Additionally, with 18 SBSs, the close proximity of SBSs results in higher interference levels, making the sleep mode particularly advantageous in reducing this interference. In the graph depicting the effect of the number of UEs, it was observed that energy-utility efficiency slightly deteriorates as the number of UEs increases.

Figure 13b compares the energy-utility efficiency of the proposed scheme with other schemes based on the number of UEs, with six SBSs and ξ=0.5. The results suggest that using a static ABS ratio and BOs can improve energy-utility efficiency compared to the ‘***Default***’ scenario. Furthermore, dynamically adjusting the ABS ratio and BOs further enhances energy-utility efficiency. The proposed scheme outperforms all other schemes. The difference between the ‘***DQN for ABS-CRE***’ and the proposed scheme indicates that incorporating SBS sleep mode, TPA, and CTV as adjustable parameters is also effective in enhancing network performance. Overall, the results indicate that the number of UEs does not significantly affect energy-utility efficiency. Instead, energy-utility efficiency is primarily influenced by the number of SBSs.

**Figure 14:**
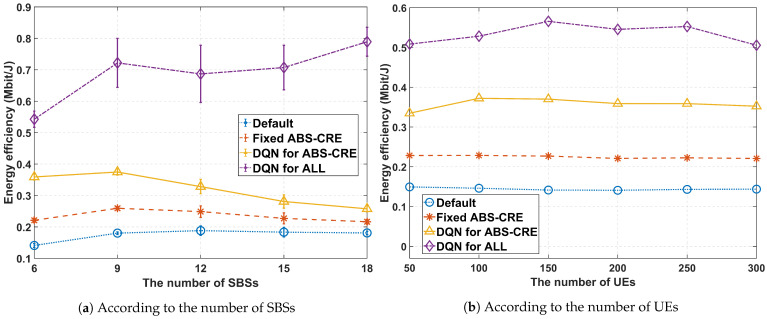
Average energy efficiency (bit/J) performance, where ξ=0.5.

Figure 14a,b depict the energy efficiency, which is defined as the network throughput divided by the network energy consumption. In other words, energy efficiency measures how much data can be transmitted per unit of energy. Figure 14a shows the results with 200 UEs deployed in the network, while Figure 14b presents the results with 6 SBSs deployed in the network. The proposed scheme enhances energy efficiency, whereas other schemes either maintain a similar level or experience a decrease as the number of SBSs increases. The sleep mode of SBSs plays a crucial role in reducing energy consumption. Deploying a large number of SBSs in the network can improve throughput, but also increases energy consumption. In Figure 14a, the graph for ‘***DQN for ABS-CRE***’ suggests that the increase in energy consumption outweighs the throughput gains as the number of SBSs increases.

**Figure 15:**
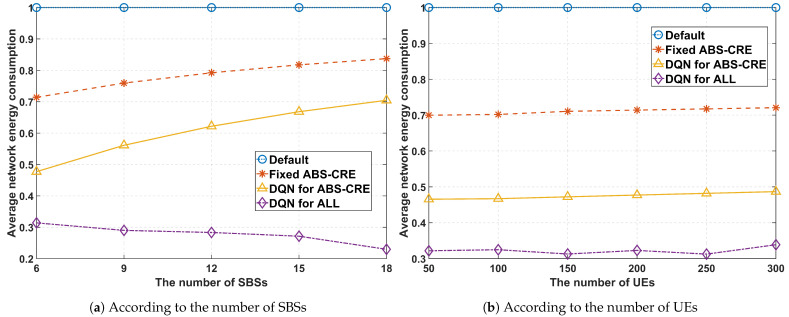
Average energy consumption performance, where ξ=0.5.

Figure 15a,b show the energy consumption relative to the ‘***Default***’ scenario. The experiment depicted in Figure 15a was conducted with 200 UEs deployed in the network and ξ=0.5, while Figure 15b presents the results with 6 SBSs deployed in the network and ξ=0.5. While using static values for the ABS ratio and BOs can reduce energy consumption by about 30%, and an adaptive approach can further improve it by approximately 50% compared to the ‘***Default***’ in the case of six SBSs, the proposed scheme achieves nearly 70% energy savings. Without sleep mode control, energy consumption increases as the number of SBSs grows. The proposed scheme effectively mitigates this by switching unnecessary or underutilized SBSs to sleep mode, thus further reducing energy consumption as the number of SBSs increases. In Figure 15b, we observe that energy consumption rises with the increasing number of UEs in the network.

**Figure 16:**
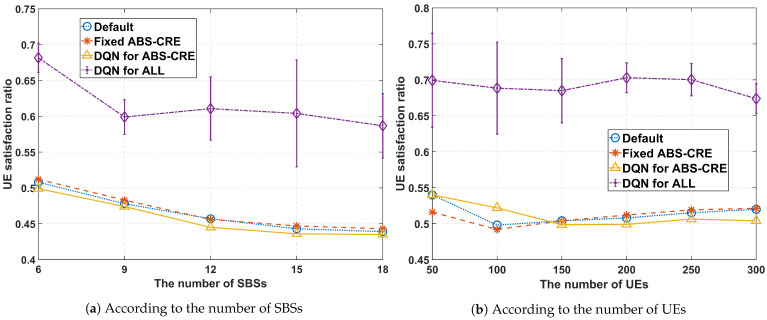
Average satisfaction ratio performance, where ξ=0.5.

Maximizing the QoS satisfaction ratio is one of our key objectives. Figure 16a,b illustrate the QoS satisfaction ratio of UEs. The experiment depicted in Figure 16a was conducted with 200 UEs deployed in the network and ξ=0.5, while Figure 16b presents the results with 6 SBSs deployed in the network and ξ=0.5. The proposed scheme enhances the QoS satisfaction ratio by approximately 15% compared to other schemes while simultaneously reducing energy consumption across all scenarios.

## 6. Discussion

The proposed framework in this paper implements DDQN as a representative DRL algorithm, but it is essential to note that our framework’s design is not algorithm-specific. The key contribution of this work lies in how we formulated the H-CRAN optimization problem into a DRL-compatible framework through carefully designed state space incorporating QoS satisfaction and load balance indicators, multidimensional action space integrating five key network parameters, and a novel reward function balancing network performance and energy efficiency.

This formulation can potentially work with various DRL algorithms such as DDPG, PPO, or TD3, as long as they can handle discrete state and action spaces. Each algorithm might offer different advantages in terms of learning stability, policy updates, and convergence characteristics. While comparing these different algorithms’ performance would be interesting and could enhance our understanding of the framework’s capabilities, such comparison would require extensive hyperparameter tuning to ensure fair evaluation. This optimization of algorithm-specific parameters, while valuable, would shift focus from the main contribution of our framework design and problem formulation.

The optimal choice of DRL algorithm would need to consider various factors including convergence speed requirements, computational resource constraints, stability needs in different network conditions, and implementation complexity in practical systems. These considerations suggest promising directions for future research, including comparative analysis of learning efficiency with different algorithms, stability evaluation under various network conditions, assessment of computational resource requirements, and investigation of implementation complexity in practical systems. Such future work could provide valuable insights into the framework’s versatility and help identify the most suitable DRL algorithms for specific deployment scenarios in H-CRAN environments.

## 7. Conclusions

We proposed a dynamic, energy-efficient eICIC scheme using deep reinforcement learning in H-CRAN, targeting improved energy-utility efficiency while maintaining QoS. Key results from the simulations show that the proposed method reduces energy consumption by up to 70% when compared to default schemes, achieving significant savings by dynamically adjusting eICIC parameters such as ABS ratios, BOs, TPA, CTV, and incorporating SBS sleep modes.

The analysis of energy-utility efficiency, depicted in the graphs, demonstrated that our approach consistently outperforms existing methods across different network scenarios, especially as the number of SBSs grows. The use of sleep mode in SBSs was particularly effective in reducing energy consumption under high interference conditions, while maintaining throughput. Additionally, energy efficiency improved with increasing energy sensitivity parameters (ξ), further validating the system’s ability to balance energy consumption with network performance.

Finally, the proposed scheme showed improved QoS satisfaction by approximately 15% compared to conventional methods, emphasizing its ability to meet service demands while optimizing energy usage. These results make the proposed scheme a strong candidate for enhancing the efficiency and sustainability of future 5G networks.

## Figures and Tables

**Table 1:** Power model parameters [17].

BS Type	$N_{\mathrm{TRX}}$	$P^{\max}$ [W]	$P^{0}$ [W]	$P^{\mathrm{sleep}}$ [W]
Wide Area	6	20	130	75
Wide Area RRH	6	20	84	56
Medium Range	2	6.3	56	39
Local Area	2	0.13	6.8	4.3

**Table 2:** Simulation parameters.

Parameters	Value
Carrier frequency	2 GHz
System bandwidth	20 MHz
Subframe duration	1 ms
The number of resource block	100
Transmission power	MBS($p_{m}^{normal}$): 20 W,
	SBS($p_{s}$): 6.3 W
Macro sector antenna pattern	$A_{H}\left(\phi \right)=-min\left[12{\left(\frac{\phi }{\phi_{3\;dB}}\right)}^{2},A_{m}\right]$,
	$A_{m}=20$ dB for 65 degrees
Cell radius	MBS: 500 m
$\alpha_{m}$	{1/10,2/10,…,9/10}
$\beta_{m}$	{0,0.05,0.1,…,0.5}
$\gamma_{s}$	{1,2,…,15}
$\delta_{s}$	{0,1,…,16}
$\epsilon_{s}$	{0,1}
Distance b/w MBS and SBS	330 m
Min. distance b/w MBS and UE	35 m
Fading block duration	100 ms
Doppler frequency	5.53 Hz
Path loss model MBS to UE	$128.1+37.6\log_{10}\left(R\right)$ dB (*R* km)
Path loss model SBS to UE	$140.7+36.7\log_{10}\left(R\right)$ dB (*R* km)
Scheduling algorithm	Round Robin (RR)
Packet size	300 Bytes
Delay QoS requirement	150 ms
Delay violation probability	0.01
Number of UEs	from 50 to 300
UE mobility speed	3 km/h
Learning rate	0.2
Discount factor	0.9
Exploration rate	1.0 diminishing 0.001 every step
Replay memory buffer	1000
Batch size	32
Macro power consumption parameters	$N_{TRX}=6,p_{m}^{0}=130$ W,
	$p_{m}^{normal}=20$ W
SBS power consumption parameters	$N_{TRX}=2,p_{s}^{0}=56$ W,
	$p_{s}=6.3$ W,
	$p_{s}^{sleep}=39$ W

## Data Availability

Data are contained within the article.

## Abbreviations

The following abbreviations are used in this manuscript:

5G	Fifth-generation
ABS	Almost Blank Subframe
AWGN	Additive White Gaussian Noise
BBU	Baseband Unit
BLER	Block Error Rate
BO	Bias Offset
CQI	Channel Quality Indicator
CRAN	Cloud Radio Access Networks
CRE	Cell Range Expansion
CTV	CQI Threshold of victim Ues
DDPG	Deep Deterministic Policy Gradient
DDQN	Double Deep Q-Network
DQN	Deep Q-Network
DRL	Deep Reinforcement Learning
EC	Effective Capacity
eICIC	Enhanced Inter-cell Interference Coordination
eMBB	Enhanced Mobile Boradband
H-CRAN	Heterogeneous CRAN
HetNet	Heterogeneous Networks
IIoT	Industrial Internet of Things
IoT	Internet of Things
LP-ABS	Low-Power Almost Blank Subframe
MBS	Macro Base Station
MCS	Modulation and Coding Scheme
MDP	Markov Decision Process
OFDMA	Othogonal Frequency-Division Multiple Access
PPO	Proximal Policy Optimzation
QoS	Quality of Service
QSI	QoS Satisfaction Indicator
RL	Reinforcement Learning
RRH	Remote Radio Head
RSRP	Reference Signal Received Power
SBS	Small Base Station
SINR	Signal-to-Interference-plus-Noise Ratio
TD3	Twin Delayed Deep Deterministic Policy Gradient
TDD	Time Division Duplex
TPA	Transmission Power of an MBS during ABS subframes
UAV	Unmanned Aerial Vehicle
UE	User Equipment
URLLC	Ultra Reliable and Low Latency Communication
ZP-ABS	Zero-Power Almost Blank Subframe
